# Growth, developmental achievements and vaccines timeliness of undocumented migrant children from Eritrea compared with Israelis

**DOI:** 10.1371/journal.pone.0193219

**Published:** 2018-03-08

**Authors:** Zohar Mor, Anat Amit Aharon, Rivka Sheffer, Haim Nehama

**Affiliations:** 1 Tel Aviv Department of Health, Tel Aviv, Israel; 2 Sackler Faculty of Medicine, School of Public Health, Tel Aviv University, Tel Aviv, Israel; 3 Sackler Faculty of Medicine, Nursing Department, Tel-Aviv University, Tel Aviv, Israel; 4 The Department of Public Health, Tel Aviv Municipality, Tel Aviv, Israel; Public Library of Science, UNITED KINGDOM

## Abstract

**Introduction:**

Israel has absorbed >40,000 Eritrean undocumented migrants since 2007, while the majority live in the southern neighborhoods of Tel-Aviv. As non-citizens and citizens infants in Israel receive free preventive treatment at the mother and child health clinics (MCHC), this study aimed to compare development and growth achievements between children of Eritrean mothers (CE) to children of Israeli mothers (CI), and assess their compliance to routine follow-up and vaccination-timeliness.

**Methods:**

This cohort study included all Israeli-born CE between 2009 and 2011, compared with a random sample of CI and treated at the same MCHC and followed-up to the age of 30-months. Dependent outcomes included anthropometric measurements, developmental achievements and adherence to immunization schedule.

**Results:**

Of all 271 CE who were compared with 293 CI, no statistically significant differences were found in birth anthropometric measurements. Yet, CE had increased weight and length than CI after reaching one year of age (*p*<0.05). CE were more likely to fail in tests assessing fine-motor skills, linguistic and socio-emotional domains than CI, while no statistical difference was found in gross-motor achievements. At the end of follow-up, 203 (74.9%) of the CE and 271 (74.1%) of the CI completed the vaccination schedule, *p* = 0.9.

**Conclusion:**

CE had greater anthropometric measurements than CI after one year of age, and showed higher impairments in fine motor, linguistic and socio-emotional domains. Adherence to vaccination was similar. The inequalities in child health should be responded in the MCTC, and Eritrean mothers should be trained with the current recommendations for child well-being.

## Introduction

Israel has faced a flux of migration from the horn of Africa since 2007, and become the destination of more than 60,000 migrants [1]. The majority (~40,000) originated in Eritrea, and more than 50% are living in Tel Aviv, especially in its southern parts [2]. According to the division of public health in Tel Aviv, 690 newborns who were born to migrants from the horn of Africa were treated in the mother and child health clinics (MCHC) across the city in 2015, while more than 95% of whom were born to Eritrean parents.

Infants until the age of 5 years in Israel are entitled to free medical follow-up and immunizations in MCHC, regardless of their legal status. Follow-up includes the assessment of the child’s well-being, anthropometrics measures which are used to define growth, nutrition, motor and psychological development. Infant’s weight, height and head circumference are measured in each visit, as low weight is strongly correlated with negative impact on child health [3,4]. Contrarily, obesity in childhood can track into adulthood and may lead to the increasing prevalence of overweight and obesity with its adverse events [5].

Children’s motor development measurements, which are classified into gross and fine motor skills, as well as cognitive, linguistic and socio emotional development achievements are all integrated, and a delay in one of the domains can strongly suspend accomplishment in another aspect [6]. The infants are expected to meet their developmental milestones in a timely manner, which are examined by the nurse during each visit to the MCHC.

Infants are immunized at the MCHC according to the child condition and the National immunization program. Pediatric vaccines have dramatically reduced the incidence of infectious disease and childhood mortality [7]. Failure to adhere with the recommended schedule may leave the children susceptible to morbidity and life threatening infectious diseases [8].

Undocumented migrants in Israel are excluded from the National medical insurance. Unlike Israeli-born children, who are entitled for medical insurance, children born to non-citizens are insured only if their parents voluntary pay a monthly fee of 40$ US. Although MCHC services are provided at no charge regardless of the legal status of the infants, migrant parents may have information gaps regarding the recommended procedures and schedule of child’s care at the MCHC. Furthermore, migrants may have established mistrust in the health system which may render them from accessing MCHC, in addition to existing language and cultural barriers [9,10].

Due to the concern that children born to undocumented migrants may have inferior health outcomes [11], this study aimed to compare development and growth achievements between infants born to Eritrean mothers to those born to Israeli mothers, and assess their compliance to routine follow-up and vaccination schedule.

## Methods

This cohort study compared anthropometric measurements, developmental achievements and adherence to immunization schedule between children who were born to Eritrean mothers and children born to Israeli mothers between 2009 and 2011.

### Participants

The study included all migrant children who were born to Eritrean mothers and treated in all the four MCHC operating in south Tel Aviv. They were compared with a random sample of children whose mothers were Israeli citizens and treated in the same MCHC.

As most of the migrants are residing in south Tel Aviv, each infant who was born to Eritrean mother was matched with another infant who was born to Israeli mother by the month and year of birth to preclude socio-economical, timeliness or geographic variation. In order to reduce possible heterogeneity and to compare vaccine adherence when the infants aged one year, only children who were living with their biological mothers and were followed-up at the MCHC for more than 12 months were included, regardless of their origin. Children whose mothers were born in Eritrea and naturalized during the follow-up period, were excluded from the study, as well as Eritrean or Israeli children who were retarded or hospitalized for more than six months. The study follow-up ended when each of the infants has reached the age of 30 months of age.

### Medical procedures at the MCHC

Routine follow-up schedule in MCHC in Israel includes nine visits up to the age of 2 years (at 2 weeks, 1, 2, 4, 6, 9, 12, 18, and 24 months). The visits include an interview with the care giver, observation of the infant’s behavior and impression from the mother and child bonding capabilities, assessment of the child’s well-being, physical examination by the doctor and the nurse and immunization, if scheduled. The summary of each visit is recorded on a standardized form in the infants’ file.

### Variables

Dependent outcomes for this study include anthropometric measurements, developmental milestone achievements, adherence to immunization schedule and blood test results performed at the age of 9 months, as were registered by the nurse. Anthropometric characteristics comprised of head circumference, height and weight which are measured during each visit at the MCHC (S1 File).

Developmental achievements are classified into four domains: gross motor, fine motor, linguistic and socio-emotional capabilities [12]. The infants’ capabilities for each domain were evaluated by different tests. For the purpose of this study, we chose one of the activities tested from each of these four domains, which were recorded throughout the different visits at the MCHC. If the infant accomplished the activity which was tested at the MCHC, or the mother reported that the infant had performed the activity at home (i.e., ability to turn at the age of six months, say three words at the age of one year, etc.), than the outcome of the test was classified as ‘success’. If the infant was unable to perform the activity, then the outcome of the test was classified as ‘failure’. All the different activities in each visit were summarized by the nurse, and in the cases in which the infant was unable to perform one or more of the activities of each domain at the nurse’s inspection room, then the entire visit was classified as ‘failure’.

Immunization adherence was tested for the third dose hepatitis B virus (HBV3), the forth diphtheria, tetanus, acellular pertussis and polio with haemophilus influenza B (DTaP-IPV4-HiB), the first measles, mumps, rubella, varicella (MMRV1) and the second hepatitis A (HAV2), which are scheduled for 6 months, 12 months (both) and 24–30 months, respectively. Vaccine timeliness was calculated by the number of days from birth to immunization date.

Infants were also expected to perform blood test to measure their hemoglobin level at the age of 9 months to detect anemia (classified as hemoglobin level lower than 11 gr/dL) caused by nutritional deficit or other health condition.

### Statistical analysis

Children born to Eritrean mothers were compared to those who were born to mothers who were Israeli citizens by maternal characteristics, gestational and birth outcomes, adherence to clinic visits and vaccinations, achievements in developmental tests and blood testes results for hemoglobin. Comparisons between continuous variables were performed by independent two-tailed Student’s *t*-tests (or the Mann Whitney test if the distribution was not normal), while categorical variables were compared by the *χ*^2^ test using SPSS (24.0 version, Chicago, IL, USA). *P* values lower than 0.05 were considered statistically significant.

Additional analysis included children who failed at least in one developmental test during the follow-up at the MCHC, who were compared to children who achieved ‘success’ in all the age-related developmental milestones at the different visits. As maternal, gestational and labor characteristics were different between Eritrean and Israeli mothers, a propensity score was calculated for infants born to Eritrean *vs*. Israeli mothers, which included maternal age, education and employment, pregnancy length and the infants’ Apgar scores after labor. In order to assess the association between maternal country of origin with the achievements in developmental tests, the multivariate model using logistic regression in the enter mode was adjusted to the propensity scores, yielding odds ratios and 95% confidence intervals.

The study was approved by the Institutional Review Board (IRB) of the Ministry of Health (permit number 1/2013, signed by the Chief Scientist). The IRB exempted the researchers from patients’ consent, as the nature of the study was retrospective and the data were collected for routine procedures at the clinic.

## Results

This cohort study included all 271 infants who were treated in MCHC in south Tel Aviv, who comprised 56.5% of all 480 Eritrean children who were treated in the city of Tel Aviv between 2009 and 2011. They were compared with a random sample of 293 Israeli children. All the children from both study arms were followed-up until they were one year of age. During the second year of the study, 64 (23.6%) of the Eritrean and 78 (26.6%) of the Israeli children were lost to follow-up, *p* = 0.4.

Eritrean mothers in this study were significantly younger compared with mother holding Israeli citizenship, had lower level of education, more commonly unemployed and their children were more likely to be medically uninsured (Table 1). Pregnancy length, child’s birth weight and head circumference at birth did not show statistically significant differences between the two groups, yet Apgar scores were lower among Eritrean children. The first visit of Eritrean children to the MCHC was delayed in an average of 18 days compared their Israeli counterparts and they had lower total number of visits, although it was not statistically significant. Generally, migrants’ children were less likely to meet development milestones than Israelis in ages 6 weeks to one year. They were more likely to fail in tests which assessed fine motoric skills, linguistic and socio-emotional domains. No statistical difference was found between migrant and Israeli children in achieving gross motor developmental milestones.

**Table 1:** Comparison between Eritrean and Israeli children treated in south Tel Aviv mother and child health clinics, 2009–2011.

	Characteristic	Eritreans N = 271	Israelis N = 293	*P*
Mothers’ characteristics	Mother’s age at infants’ birth^ (mean±Sd in years)	29.3±4.6	34.3±5.8	<0.001
	Mother education <8 years	128 (73.1)	5 (1.7)	<0.01
	Mother is unemployed	151 (74.4)	75 (26.4)	<0.01
	Medically insured infant	52 (20.0)	284 (99.3)	<0.01
Pregnancy and birth outcomes of the infants	Pregnancy length^ (mean±Sd in weeks)	39.1±2.0	38.9±1.8	0.2
	Birth weight^ (mean±Sd in Kg)	3.2±0.5	3.1±0.5	0.7
	Apgar 1 minute^ (mean±Sd)^#^	8.6±1.3	8.8±0.8	0.05
	Apgar 5 minutes^ (mean±Sd)^#^	9.7±0.9	9.9±0.5	0.02
	Birth head circumference^ (mean±Sd in cm)	34.6±1.6	34.1±1.7	0.9
	Male sex	125 (50.8)	137 (48.1)	0.5
	Infant was detected with a chronic disease	67 (25.2)	62 (21.3)	0.3
First visit to clinic^#^ (median days, 25–75 IQR in days)		40, 32–54	22, 11–34	<0.01
Total number of visits to clinic during 24 months^ (mean±Sd)		8.2±1.8	8.6±2.3	0.06
Fails 6 weeks tests	Holds the head up when lying on the stomach	15 (6.3)	9 (3.4)	0.1
	Turn his head toward sounds^$^	16 (6.7)	6 (2.3)	0.01
	Gurgle sound	37 (15.7)	22 (8.5)	0.01
	Smiles	28 (15.8)	24 (9.0)	0.01
	Failure summary at 6 weeks	79 (33.2)	56 (21.1)	<0.01
Fails 6 months tests	Holds the head and chest up when lying on the stomach	20 (8.3)	17 (6.6)	0.5
	Turn his head towards sounds	37 (15.3)	44 (17.1)	0.6
	Passes objects between hands^$^	27 (11.2)	12 (4.7)	0.007
	Makes sounds	44 (18.2)	19 (7.3)	<0.001
	Responds to strangers	30 (12.4)	16 (6.2)	0.02
	Failure summary at 6 months	65 (27.0)	68 (26.3)	0.9
Fails 12 months tests	Walks with assistance	19 (7.7)	24 (9.9)	0.4
	Talks 1–2 words	95 (38.6)	57 (23.6)	<0.001
	Identifies and points at objects^$^	68 (27.6)	21 (8.7)	<0.001
	Returns a ball	96 (39.0)	21 (8.7)	<0.001
	Names body part	155 (63.0)	68 (28.1)	<0.001
	Failure summary at 12 months	159 (64.6)	84 (34.7)	<0.001
Fails 24 months tests	Runs without falling	7 (3.4)	6 (4.2)	0.8
	Climbs stairs independently	10 (4.9)	9 (6.4)	0.6
	Draws a circle line^$^	80 (39.0)	16 (11.3)	<0.001
	Plural/single differentiation	164 (80.0)	85 (60.3)	<0.001
	Composes a sentence of more than 3 words	163 (79.5)	76 (53.9)	<0.001
	Toilet trained	175 (85.4)	102 (72.3)	0.004
	Failure summary at 24 months	177 (86.3)	118 (82.7)	0.5
Blood tests results	Performed blood test	155 (57.4)	246 (84.8)	<0.001
	Hemoglobin ^ (mean±Sd in gr/dL)	11.8±0.9	11.7±0.8	0.5
	MCV^ (mean±Sd in femoliter/cell)	77.4±4.1	74.9±4.3	<0.001
Vaccination date (from birth until immunization)	HBV 3^#^ (median days, 25–75 IQR)	255 (203–270)	203 (189–241)	0.2
	MMR^#^ (median days, 25–75 IQR)	380 (370–408)	385 (374–416)	0.4
	DTaP-IPV-HiB-4^#^ (median days, 25–75 IQR)	419 (391–547)	402 (380–457)	0.1
	HAV 2^#^ (median days, 25–75 IQR)	801 (756–865)	764 (740–811)	0.02

^ Student’s *t*-test

^#^ Mann Whitney test

Sd- standard deviation

MCV- Mean corpuscular volume

Kg- Kilograms

cm- centimeters

IQR- intra quartile range

^$^ Fine motor qualifications

Eritrean mothers were less likely to perform hemoglobin tests to their children than mothers holding Israeli citizenship. Yet, infants’ hemoglobin levels at 9 months in those tested did not show statistical significant differences with those who were born to Israeli citizens, although the children migrants’ mean capsular volume (MCV) was higher.

By the end of the study follow-up when the infants become 30 months of age, 203 (74.9%) of the migrant children and 217 (74.1%) of the Israelis have completed the vaccination schedule, *p* = 0.9. No statistical differences were found in the timeliness of vaccination between migrants and Israelis, excluding the second vaccine of HAV, which was performed later among the migrants (*p* = 0.02).

**Fig 1:**
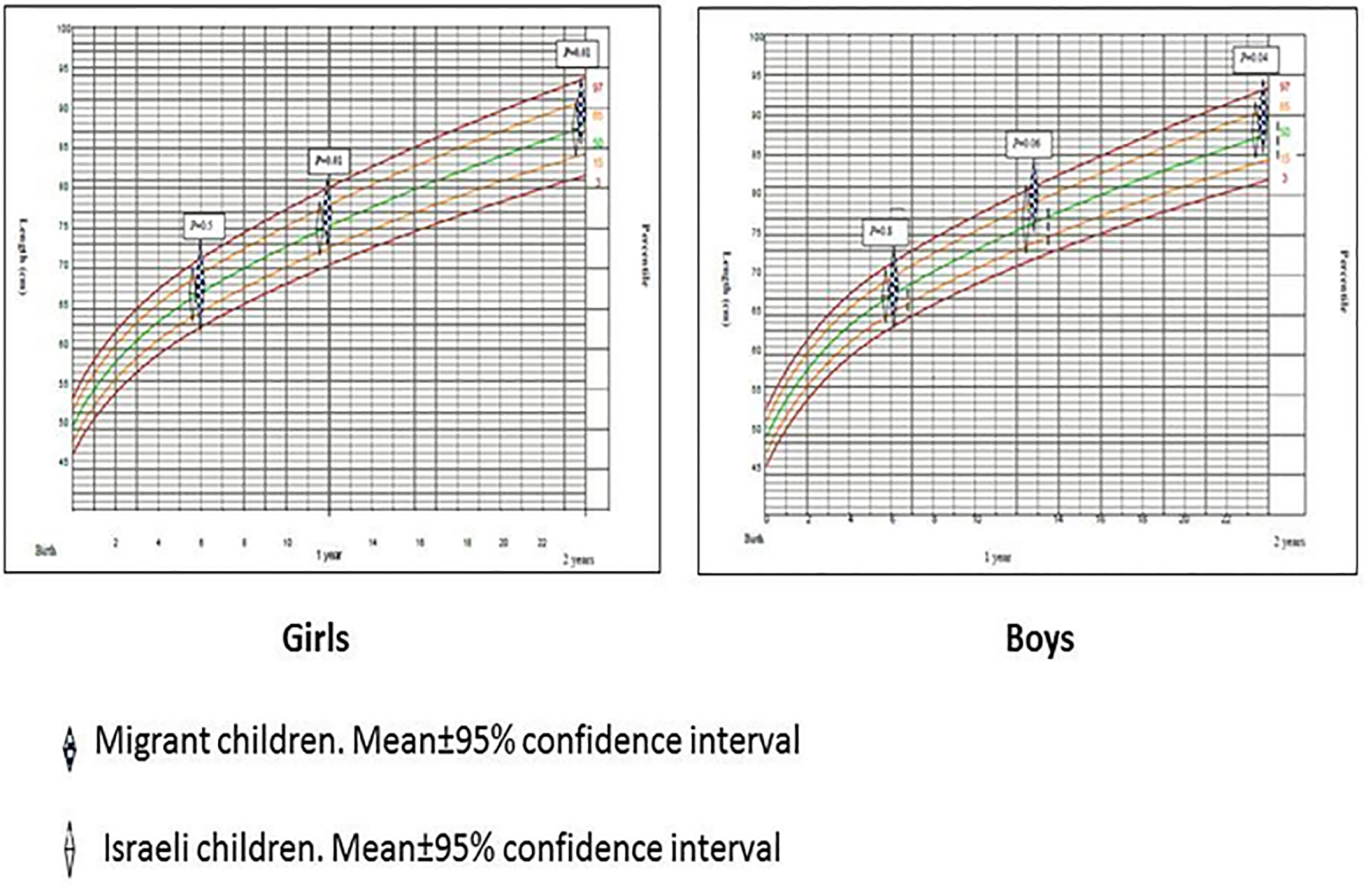
Length development curves of migrant and Israeli children, birth until 2 years.

**Fig 2:**
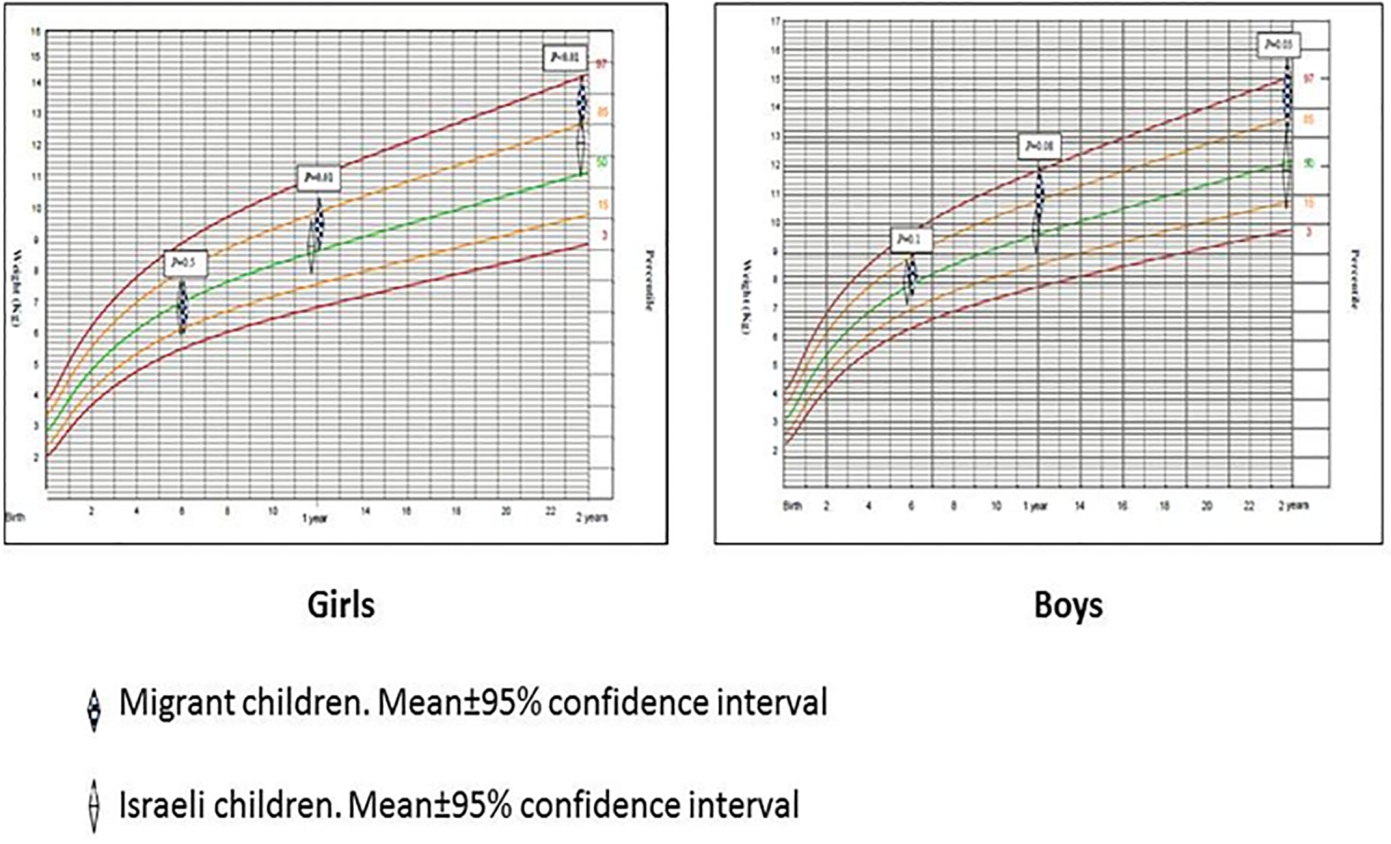
Weight development curves of migrant and Israeli children, birth until 2 years.

**Fig 3:**
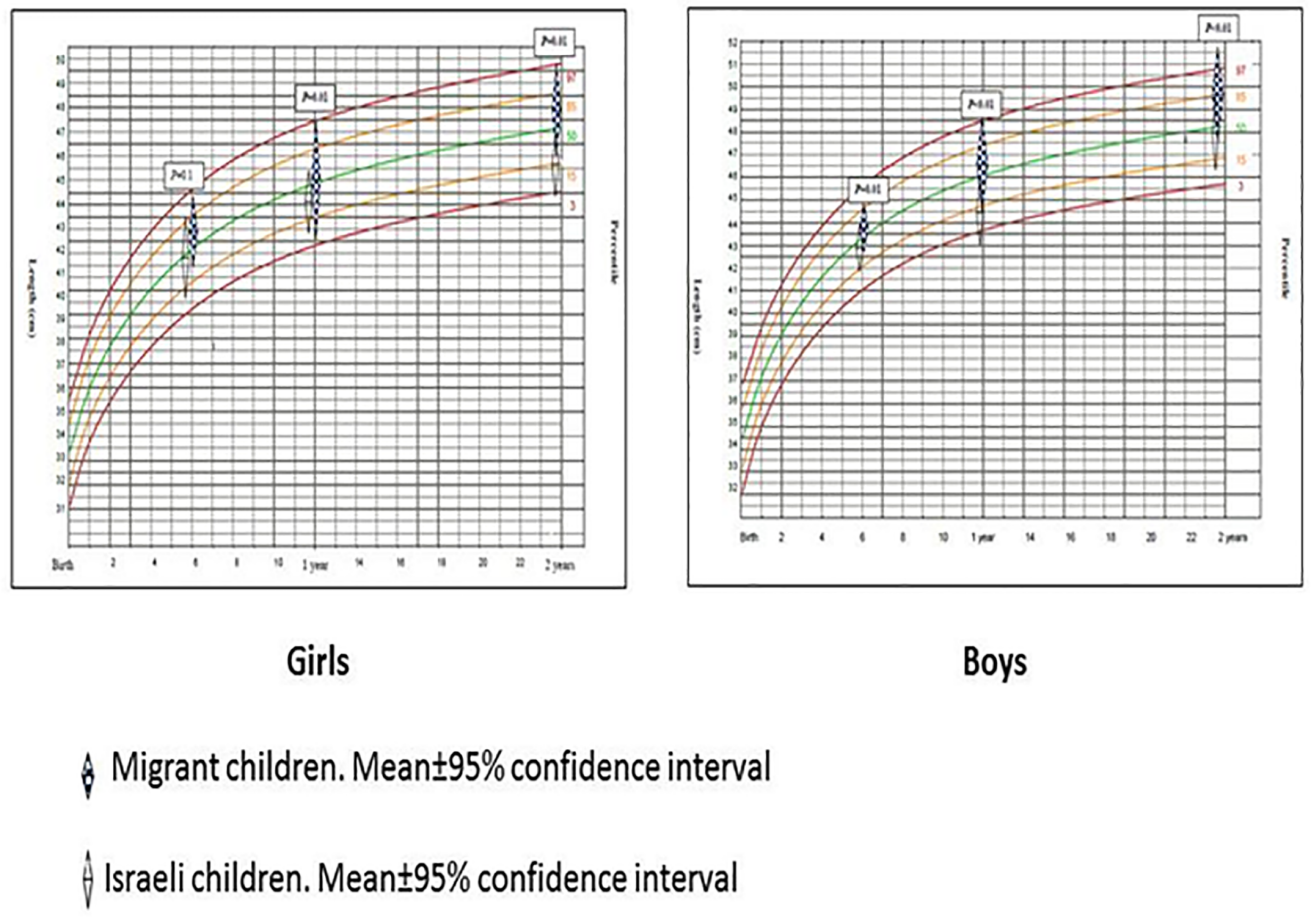
Head circumference development curves of migrant and Israeli children, birth until 2 years.

Migrant children were not different than Israeli in their anthropometric characteristics measured at birth (length, weight and head circumference, both in boys and girls), while all were at approximately 50% percentile. However, from 6 months and after, the gap in all anthropometric measurements between Eritrean and Israeli children has gradually increased. Since one year of age all anthropometric measurements among children born to Eritrean mothers reached at ~85% percentiles, while infants born to mothers holding Israeli citizenship remained in the 50% percentile, *p*<0.05 (Figs 1–3).

Table 2 includes the comparison between infants who failed in at least one developmental test with those who succeed in all tests. Children who were born Eritrean mothers, or to mothers who were less educated or unemployed were less likely to pass all tests. In addition, infants who failed in at least one developmental test had lower Apgar scores at 5 minutes after birth.

**Table 2:** Comparison of children who failed in at least one test which examines developmental milestones to those succeed in all test in all visits.

	Characteristic	Failed test in at least one visit N = 359 (%)	Passed all tests N = 192 (%)	*P*
Type of analysis		Univariate analysis		
Mother details	Mother of Eritrean origin	205 (57.1)	62 (32.3)	<0.001
	Mother’s age at infants’ birth^ (mean±Sd in years)	31.9±6.0	32.2±5.5	0.5
	Mother education <8 years	99 (35.2)	31 (18.2)	<0.001
	Mother is unemployed	154 (50.3)	65 (38.5)	0.02
	Medically insured infant	95 (27.5)	20 (10.6)	<0.001
Pregnancy and birth	Pregnancy length^ (mean±Sd in weeks)	38.9±2.0	39.2±1.6	0.02
	Apgar 1 minute^#^ (mean±Sd)	8.7±1.1	8.8±0.7	0.2
	Apgar 5 minutes^#^ (mean±Sd)	9.7±0.8	9.9±0.4	0.02
	Birth weight^ (mean±Sd in Kg)	3.1±0.5	3.2±0.5	0.006
	Birth head circumference^ (mean±Sd in cm)	34.3±1.8	34.3±1.3	0.8
	Male sex	174 (51.6)	86 (47.3)	0.4
	Infant was detected with a chronic disease	95 (26.8)	31 (16.3)	0.006
Weight-length percentiles in different visits	Percentile at 6 months^#^ (median, 25–75 IQR)	42 (21–72)	40 (21–64)	0.5
	Percentile at 12 months^#^ (median, 25–75 IQR)	61 (34–82)	65 (37–85)	0.06
	Percentile at 24 months# (median, 25–75 IQR)	70 (46–91)	64 (37–84)	0.5

^ Student’s *t*-test

^#^ Mann Whitney test

Sd- standard deviation

MCV- Mean corpuscular volume

Kg- Kilograms

cm- centimeters

IQR- intra quartile range

Propensity scores of maternal and labor characteristics of Eritrean and Israeli mothers were 0.13±0.11 and 0.76±0.34 respectively, *p*<0.001. In the multivariate analysis, been born to Eritrean mother was associated with failure in one or more developmental test, after adjusting to maternal and labor characteristics (Table 3).

**Table 3:** Multivariate analysis associating maternal characteristics with infants’ achievements in developmental tests.

Characteristic	Odds ratio (95% confidence interval)	*P*
Mother was born in Eritrea	2.7 (1.3–5.6)	0.01
Propensity scores	0.9 (0.4–2.4)	0.9

## Discussion

Infants born to Eritrean mothers had greater anthropometric measurements than those who were born to Israelis citizens after 6 months of age, and the gap has gradually increased with age. Gross motoric skills were similar in migrants and Israelis infants, but the achievements of infants born to Eritrean mothers were inferior to infants born to Israeli mothers in fine motor, linguistic and socio-emotional domains.

The gap in anthropometric measurements may reflect genetic and intergenerational differences between the Eritrean and Israeli children. Other European countries [13], such as Switzerland [14], Austria [15] and the Netherlands [16] have also reported similar findings between migrant children compared with the local counterparts. Additionally, Eritrean mothers may have enjoyed the "healthy migrant effect" [17] contributing to better health status and also more favorable pregnancy and birth outcomes compared to the Israeli counterparts [18]. Migrant mothers are usually healthier than mothers who are citizens of the hosting country, and therefore have lower rates of preterm, low birth weight and infant mortality [19]. It is also plausible that the gap difference is associated with different eating patterns of the migrants, variations in nutrition habits and cultural norms that do not recognize overweight as a problem [16]. Although children born to Eritrean mothers in our study showed higher height and weight dimensions than children born to Israeli mothers, their measurements were found within the normal growth margins. In order to maintain normal weight, Eritrean mothers should be aware that overweight and obesity are associated with adverse health consequences, such as cardiovascular diseases, diabetes mellitus and cancer upon maturation [5]. Furthermore, children’s overweight can affect self-esteem and influence their social and cognitive development [14].

Migrant women may find it financially demanding to extend unpaid maternity leave or may not have the option to obtain a flexible work schedule. They are therefore required to return work soon after labor, and discontinue lactating and use a substitute for human milk [16,20]. The nurses at the MCTC in our study reported that Eritrean mothers tend to feed their infants with fortified milk powders rather than using the recommended (and also more expensive) formulas, which include the optimal nutrients. These milk powders are marketed in containers similar in shape, size and color to the recommended formulas, somehow confusing to inexperienced customers. Nurses at the MCHC also commented that Eritrean mothers tend to feed their children with snacks or sweet drinks in greater quantities than Israeli citizens mothers.

Migrant children showed similar accomplishments in gross motor functions, which are generally associated with universal child’s growth. Yet, their achievements in developmental domains, which require infants’ stimulation such as fine motor, linguistic and socio-emotional aspects, were inferior to that of Israelis. Lower level of fine motor performance was also found in migrant children in Tyrol, Austria [15] and in linguistic and socio-emotional domains in Bavaria, Germany [21]. It is either that the care givers were not provided the sufficient parental training or that they lack leisure time to spend with their children. Eritrean migrates in Israel are not legally permitted to work in Israel, and may be employed unofficially, while their income is low. In order to support their siblings, both parents are required to work for long hours instead of spending leisure time with their children, usually leaving them in unofficial nurseries. Theses nurseries are usually operated by inexperience women in a crowded, under-equipped and frequently lack hygienic compounds and the infants may be left in playpens for long hours. The women who are in charge generally cannot provide the infants with the adequate attention, nor can they deliver the recommended stimulations to the children which are required for motoric, cognitive and emotional development. As a result, linguistic and socio-emotional gaps between infants born to Eritrean mothers and children born to Israeli citizens have increased with age.

Migration is associated with specific life conditions and challenges that can impact health. Loss of family and friends, difficulties in acculturation and experiencing negative post-migration are likely to play a role in increased risk of mental health disorder and even post-natal depression [22,23]. Young Eritrean mothers are away from their family and may lack advices from older relatives who may guide them in parenthood and addressing children well-being. This situation underlines the role of the nurses who are working in the MCHC in advising mothers of foreign origin with the recommended dietary intake and counselling them regarding the appropriate motoric and cognitive child’s stimulation. The nurses should also confirm that the mothers are aware of the recommended appointments schedule and also remind them before the next scheduled visit. Working hours of MCHC may be adjusted to the tight schedule of the working parents, and staff should be trained to deliver cultural competent health messages.

Although it is the first study compared growth and development outcomes between Eritrean and Israeli children, it may be subject to several methodological limitations. First, the study may not represent the entire migrant population in Israel. Nevertheless, it is estimated that 75% of all children born to Eritrean parents were living in Tel Aviv [2]. An additional selection bias is that not all migrants used the MCHC services, although the service is free of charge, universal and accepted by the Eritrean community. Third, due to linguistic barriers between the mothers and the nurses, they may have not completely understood the tests performed at the MCHC. Yet, most of the tests were conducted in objective manner by the nurse at the MCHC while using standardized methods. Lastly, excluding infants who did not complete 12 months of follow-up precluded the possibility to calculate vaccine coverage rate or compare the entire Eritrean cohort.

In summary, this study demonstrated that first generation Eritrean migrant children had greater anthropometric measurements than Israelis and had higher prevalence of impairment in fine motor, linguistic and socio-emotional domains compared with their Israeli counterparts. Children born the Eritrean mothers had lower number of visits to the MCHC, but their adherence to vaccination was not different to that of children born the Israeli citizens. The inequalities in child health should be responded by the health system, and the staff who works at MCHC should train mothers of foreign origin in the current recommendation for child well-being.

## Supporting information

S1 File Data for Plos One.pdf. (XLS)
